# Mechanisms for the formation of active sites in single-atom alloys

**DOI:** 10.1039/d5nr05517b

**Published:** 2026-04-13

**Authors:** Ioannis Karageorgiou, Angelos Michaelides, Fabian Berger

**Affiliations:** a Yusuf Hamied Department of Chemistry, University of Cambridge CB2 1EW Cambridge UK am452@cam.ac.uk fb593@cam.ac.uk

## Abstract

Single-atom alloys (SAAs) show great promise in heterogeneous catalysis, yet their synthesis remains challenging. To address this challenge, we elucidate the fundamental surface mechanisms by which dopant adatoms deposited on Cu and Ag surfaces become embedded active sites in the host metal. Using density functional theory, we identify periodic trends across the transition metal (TM) series. Adatoms diffuse nearly freely across terraces due to very low diffusion barriers, whereas direct incorporation into terraces is unfavourable. In line with conventional wisdom, step edges and kink sites facilitate dopant incorporation, confirming their critical role in alloy formation. Attachment of adatoms to steps and kinks from the lower terrace is energetically favoured, and incorporation proceeds either from this attached state or when adatoms approach a step edge from above, where reactions frequently occur without an activation barrier. Incorporation barriers are generally lowest for early and central TMs, increase towards late TMs, and are slightly higher on Cu than on Ag surfaces. Our simulations further reveal how embedded dopants influence the behaviour of diffusing adatoms. They rationalise experimental observations for Pd on Cu, where repulsive adatom–dopant interactions repel diffusing adatoms from dopant-rich regions near steps, suppressing otherwise dominant incorporation pathways, and predict dopant-anchored adatom islands for attractive elements such as Ru on Cu. Overall, this work provides a unified perspective on how specific surface sites and adatom–dopant interactions govern dopant incorporation, offering guidance on the surface environments most conducive to SAA formation across different dopant elements.

## Abstract 2

Active site formation in single-atom alloys is governed by defects such as steps and kinks and exhibits periodic trends across the transition metal series. Dopant–adatom interactions further control adatom mobility and incorporation pathways.

## Graphical abstract

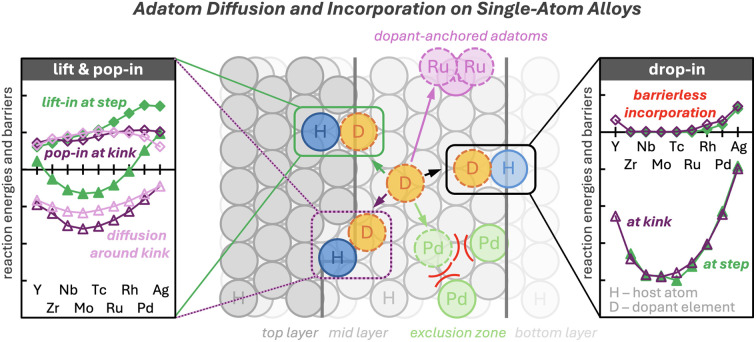

## Introduction

Heterogeneous catalysis drives most industrial chemical processes,^1,2^ but conventional solid catalysts are often constrained by linear scaling relations, resulting in a trade-off between activity and selectivity.^3–6^ Single-atom alloys (SAAs), in which atomically dispersed reactive dopants are embedded in relatively inert host metals, can overcome this limitation.^7–12^ Their architecture can enable spatial separation of active reaction sites from inert desorption sites, allowing products to spill over and thereby introducing a beneficial bifunctionality.^9,13^ Together with the unique free-atom-like electronic structure of dopant sites, SAAs can enhance both activity and selectivity.^14–18^ With tunable composition and low dopant concentrations, typically only a few percent in the surface layer, SAAs also maximize the efficiency of costly dopant metals, making them a highly promising class of solid catalysts.

As SAAs are exciting catalysts, most experimental and computational studies focus on their catalytic performance, using samples and models with already formed active sites consisting of embedded dopants.^7,9,17,19–25^ The interplay between theory and experiment has been leveraged with great success for selective hydrogenation,^8,26^ selective oxidation,^27^ dehydrogenation,^28^ and CO oxidation.^29^ Much less attention, however, has been devoted to understanding how these active dopant sites form in the first place.^30–32^ Elucidating this process is not only of fundamental mechanistic interest but could also inform improved synthesis strategies, which remain a major bottleneck due to experimental challenges.^33–35^

Among the methods available for synthesising highly dilute metal alloys,^7,36–39^ physical vapor deposition (PVD) is widely employed, particularly for well-defined single crystal surfaces.^40–43^ In this work, we model conditions representative of PVD on Cu and Ag surfaces. However, other synthesis techniques exist that can lead to the formation of supported nanoparticle catalysts, which represent an important realisation of SAAs. Such systems may be synthesised under conditions not captured by the present models, for example *via* galvanic replacement,^7,36^ sequential reduction,^7,44,45^ or co-impregnation.^7,46^ Our focus is on establishing atomistic incorporation mechanisms and trends under vacuum and surface science conditions, thereby providing a fundamental mechanistic understanding of SAA formation upon which future studies of more complex environments can build.

The synthesis of SAAs investigated here typically begins with the deposition of a trace amount of dopant onto the host metal surface, initially forming adatoms that reside on the surface before becoming incorporated into the alloy. From this initial state, several processes can occur, which we elucidate and relate to experimental and computational studies.^31,43,47–50^ Mechanisms for the diffusion, attachment, and incorporation of adatoms into metal surfaces have been investigated previously,^30–32,48–64^ but typically only for very few, specific combinations of adatom and host elements and often focusing on a limited subset of possible pathways. As a result, a broader, unified picture of how adatoms evolve into embedded dopants and how these mechanisms vary across the periodic table has remained incomplete.

Using spin-restricted density functional theory (DFT) with the optB86b-vdW exchange–correlation functional,^65^ we investigate the series of 4d transition metal (TM) dopants on Cu and Ag host surfaces. In addition to the ideal and commonly studied (111) terrace, we model step edges using the (211) and (322) surfaces and construct additional models for kink defects derived from these facets. The considered step models correspond to one of the two step edge types found on a (111) surface and are the most widely studied.^32,50,59,60,62,64,66^ By treating all relevant pathways within a single, consistent computational framework, we establish a coherent mechanistic understanding of dopant incorporation and obtain directly comparable energetics, enabling the identification of robust periodic trends across dopant elements and assessment of host–metal effects. We also show how already embedded dopants influence the behaviour of subsequently deposited adatoms, and consequently the formation of active sites. Our findings align with recent observations that dopants modify the mobility of undercoordinated metal atoms,^67^ suggesting broader implications for catalyst stability and surface restructuring under reaction conditions.

## Results and discussion

**Fig. 1:**
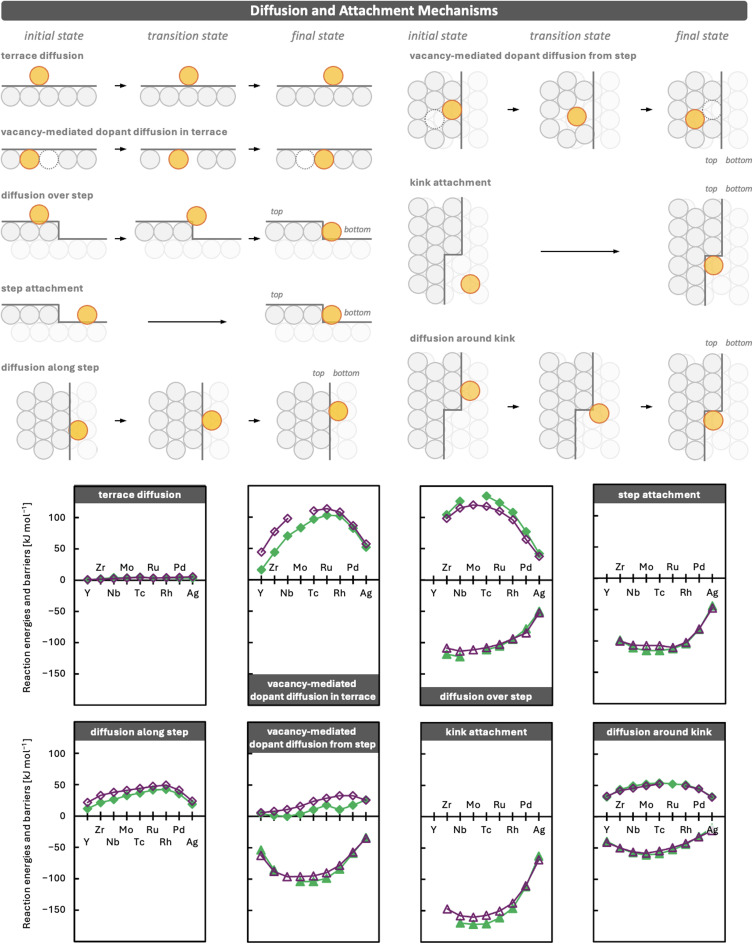
Schematic overview of diffusion and attachment mechanisms considered in this work, together with periodic trends in reaction energies (triangles) and barriers (diamonds) for 4d TM adatoms on Cu (green) and Ag (purple) surfaces. Investigated mechanisms are: *terrace diffusion*, *vacancy-mediated dopant diffusion in terrace*, *diffusion over step*, *step attachment*, *diffusion along step*, *vacancy-mediated dopant diffusion from step*, *kink attachment*, and *diffusion around kink*. Hetero adatoms and dopants are shown in orange, host atoms in grey (upper layer) and light grey (lower layer), and vacancies as dotted circles. Numerical values are provided in Section S2 of the SI. Connecting lines are shown as guides to the eye. Missing data points indicate convergence issues in locating the corresponding stationary points.

An adatom deposited on a surface can diffuse across the terrace, attach to defects such as step edges or kink sites, and eventually become incorporated into the surface. We first discuss the diffusion and attachment processes shown in Fig. 1, as these determine how adatoms move across the surface and reach defects. We then investigate the different incorporation mechanisms on terraces, step edges, and kinks and identify the trends that govern their energetics. Finally, we combine these insights to determine which reaction pathways dominate and illustrate how already embedded dopants can influence adatom diffusion and incorporation. Full computational details are provided in Section S1 of the Supplementary Information (SI), including a description of the transition state search procedure using the climbing image nudged elastic band (CI-NEB)^68,69^ method and the dimer method.^70^

### Facile terrace diffusion and trapping at step edges and kinks

Starting from an adatom deposited on a terrace site, diffusion across the terrace is extremely facile. This behaviour is consistent with previous studies on *terrace diffusion* of adatoms.^31,50–52,71^ The energy differences between adsorption sites and the corresponding diffusion barriers are generally predicted to be below 5 kJ mol^−1^. Such small barriers enable adatoms to rapidly explore large regions of the terrace. Relative adsorption energies and diffusion barriers are provided in Section S2 of the SI.

Step edges separate an upper and a lower terrace and can therefore be approached by adatoms from either side. Kink sites correspond to local irregularities along a step edge, formed where the step advances or retreats by a single atomic row. These structural features play a central role in guiding adatom motion and in determining how adatoms attach to defects or become incorporated into the surface.

When an adatom approaches a step edge from the upper terrace, it may diffuse over the step and attach from the lower terrace.^48,49,56,59^ This process is associated with comparatively large Ehrlich–Schwoebel barriers.^61,72,73^ We find that these barriers display an inverse U-shaped periodic trend (Fig. 1) with the dopant element. Because the adatom loses coordination in the transition state, elements that form stronger bonds with the host show higher barriers, in some cases far exceeding 100 kJ mol^−1^. Very late TMs, which form weaker bonds with the host,^17^ exhibit smaller barriers, as low as about 40 kJ mol^−1^ for Ag adatoms, which may be surmountable at moderate temperatures.

Adatoms approaching a step edge from the lower terrace readily attach to the ascending step.^48,49^ The barriers for this attachment do not exceed those for terrace diffusion and can become barrierless once the adatom is close to the step.^48,49^ Since attachment increases the coordination of the adatom, it is strongly exothermic, releasing up to 115 kJ mol^−1^ for Mo on Cu. The exothermicity decreases along the dopant series (triangles), reaching 44 kJ mol^−1^ for Ag on Cu, consistent with the weaker bonds formed by late TMs.

Once an adatom is attached to a step edge on the lower terrace, it may diffuse along the step.^55,56^ The barriers for this process are much higher than those for terrace diffusion, reaching up to about 50 kJ mol^−1^ for Rh on Ag. As a result, diffusion along step edges is comparatively slow. At elevated temperatures relevant for catalysis, these barriers may be overcome, allowing adatoms to migrate along the step until they encounter a kink site. The barriers are smaller for late TMs. For the coinage host metal adatoms Cu and Ag, step edge diffusion barriers are only about 30 and 20 kJ mol^−1^, respectively, which provides a possible mechanism for the growth of step edges. An adatom diffusing along a step and approaching a kink can either diffuse around the kink, with barriers slightly above 50 kJ mol^−1^ for central TMs and lower barriers for early and late TMs, such as 31 kJ mol^−1^ for Y and 22 kJ mol^−1^ for the highly mobile Ag adatoms on Cu. However, as discussed in the next section, the *near-kink pop-in* incorporation mechanism competes with this diffusion pathway and can also contribute to step growth.

When an adatom approaches a kink from the lower terrace, it attaches strongly.^19,62^ Similar to attachment at a step edge, the barriers for this process do not exceed those for terrace diffusion^48^ and can become barrierless once the adatom is close to the kink. Because kink sites offer the largest number of neighbouring host atoms for an adatom, attachment is highly exothermic, releasing up to 172 kJ mol^−1^ for Mo on Cu. The stabilisation decreases along the dopant series as the strength of the bonds formed between the adatom and the host atoms reduces, and eventually reaches 63 kJ mol^−1^ for Ag on Cu. Overall, and in line with the literature, the stronger binding at kink sites compared with straight step edges reflects the increased coordination. Adatoms therefore preferentially occupy sites that maximise the number of neighbouring host atoms: from terraces, where they interact only with atoms beneath, to step edges, where they gain additional neighbours on one side, and finally to kinks, where they interact with host atoms from two sides. This preference is more pronounced for elements that form stronger bonds with the host.

### Adatom incorporation mechanisms at terraces, steps, and kinks

**Fig. 2:**
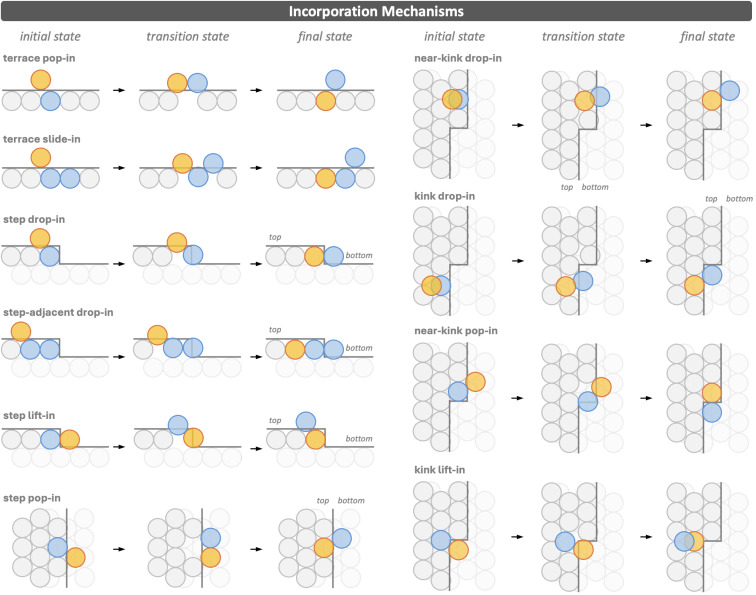
Schematic illustration of the incorporation mechanisms considered in this work. Shown are the reactant, transition state, and product structures for: *terrace pop-in*, *terrace slide-in*, *step drop-in*, *step-adjacent drop-in*, *step lift-in*, *step pop-in*, *near-kink drop-in*, *kink drop-in*, *near-kink pop-in*, and *kink lift-in*. Hetero adatoms and dopants are shown in orange, moving host atoms in blue, and host atoms in grey (upper layer) and light grey (lower layer). Simulation cell sizes are provided in Section S1 of the SI.

Once an adatom has diffused across the surface and interacted with step edges or kink sites, it may become incorporated into the lattice. The incorporation mechanisms considered in this work are illustrated in Fig. 2. These processes embed the adatom into the surface and expel a host atom. Depending on where this expelled atom is located, it may remain attached to the ascending step edge or kink, or it may start diffusing, or undergo a consecutive incorporation when expelled onto the upper terrace.

We first qualitatively introduce the mechanisms considered and then discuss the observed trends. On terraces, the adatom (orange) may directly replace a host atom in the surface layer,^31,32,53^ expelling it as a new adatom, following the *terrace pop-in* mechanism. Alternatively, incorporation can proceed through a concerted motion of the adatom together with two host atoms,^32,54^ in which the adatom slides into the terrace while the surrounding host atoms rearrange, alleviating steric constraints associated with direct exchange. In both cases, the adatom becomes embedded in the terrace, while the expelled host atom is left on the surface: in the former case directly attached to the newly incorporated dopant, and in the latter bonded to neighbouring host atoms.

When an adatom approaches a step edge from the upper terrace, incorporation can occur directly at the step. In the *step drop-in* mechanism, the adatom replaces a host atom in the step edge, which is expelled onto the lower terrace as a new adatom. This mechanism has been the subject of several studies.^32,48,49,56,59–62^ The *step-adjacent drop-in* mechanism, which has attracted considerably less attention and has so far only been investigated for the (100) facet,^63^ places the dopant in the row adjacent to the step and likewise expels a host atom onto the lower terrace. This process proceeds through a concerted motion of the adatom and two host atoms. We also attempted to identify a concerted mechanism involving the adatom and three host atoms. However, this was often unsuccessful; instead, the reaction pathway separates into two steps, namely a *terrace slide-in* followed by a *step-adjacent drop-in*, suggesting that concerted incorporation is effectively limited to the dopant and at most two host atoms. Both identified incorporation pathways embed the dopant in the upper terrace and create a new kink site, with the expelled host atom attached to the step from below.

Adatoms that are attached to a step edge on the lower terrace can incorporate into the step through two pathways. In the *step lift-in* mechanism, also discussed in previous work,^32,57,58^ the attached adatom replaces a host atom in the step edge and expels it onto the upper terrace, where it becomes a new adatom. Following the *step pop-in* mechanism, the adatom again replaces a host atom, but the expelled atom remains on the lower terrace,^55^ where it forms a new kink site. In both cases, the dopant ends up embedded in the step edge, while the expelled host atom either becomes an adatom on the upper terrace or forms a kink protruding from the step edge on the lower terrace.

Kink sites are widely recognised as particularly important locations for adatom incorporation because of their low coordination and high reactivity.^58,61,62,64^ Building on this, we consider four incorporation pathways at kink sites, which differ depending on the precise position of the adatom relative to the kink. When the adatom is located on the upper terrace adjacent to the kink, incorporation can proceed *via* the *near-kink drop-in* mechanism. In this case, the adatom replaces a host atom in the step edge next to the kink and expels it onto the lower terrace, thereby creating an additional kink that can serve as a starting point for the growth of a new row of atoms. If the adatom instead incorporates directly at the kink site, the process follows the *kink drop-in* mechanism. Here, the expelled host atom also moves to the lower terrace, but the kink position shifts by one lattice site along the step edge, effectively propagating the step. On the lower terrace, a dopant attached to a step next to a kink can insert into the step and displace the host atom forming the kink, growing the step by one atom *via* the *near-kink pop-in* mechanism. The *kink lift-in* mechanism begins with the adatom attached to the kink and lifts a fully coordinated host atom onto the upper terrace. All four pathways embed the dopant in the upper terrace near the kink, yet they lead to distinct structural outcomes: *near-kink drop-in* creates a new kink; *kink drop-in* and *near-kink pop-in* advance the step without generating additional kinks; and *kink lift-in* produces a host adatom on the upper terrace.

### Adatom size and bonding strength drive periodic trends in incorporation barriers and reaction energies

**Fig. 3:**
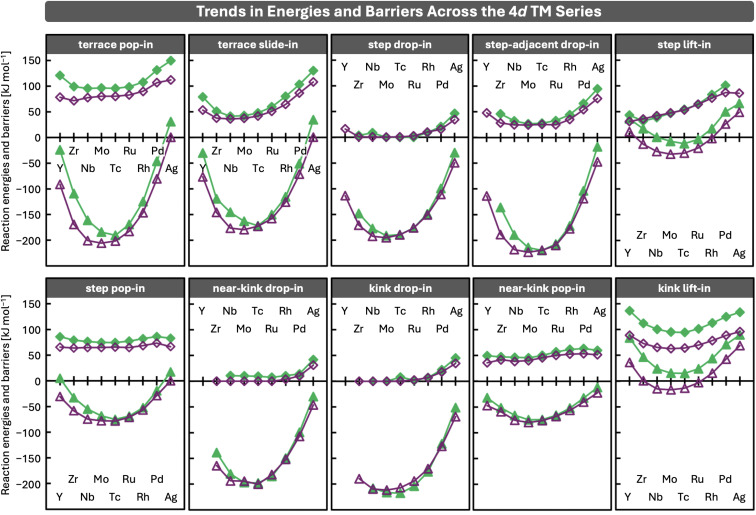
Periodic trends in reaction energies (triangles) and barriers (diamonds) for incorporation of 4d transition metal dopants on Cu (green) and Ag (purple) surfaces. Energies are given in kJ mol^−1^. Reaction energies exhibit U-shaped trends across the dopant series, whereas reaction barriers show weaker or no clear periodic dependence. Numerical values are provided in Section S2 of the SI. Connecting lines are shown as guides to the eye. Missing data points indicate convergence issues in locating the corresponding stationary points.

With the mechanistic picture in place, we next consider the energetics of the pathways. Fig. 3 summarises the reaction energies (triangles) and barriers (diamonds) associated with the incorporation mechanisms discussed above, revealing clear periodic trends across the 4d TM series. These trends arise from variations in atomic size and bonding strength along the series: central TMs are similar in size to the host but considerably more reactive, gaining substantial stabilisation upon incorporation; early TMs are larger, introducing a size mismatch that reduces the stabilisation gained; and late TMs form weaker bonds with host atoms,^17^ and therefore exhibit less exothermic reactions and typically higher incorporation barriers.

The key factor underlying the periodic trends in reaction energies is the change in coordination experienced by the adatom during incorporation. Among all surface sites, an atom embedded in a terrace has the largest number of direct neighbours, followed by incorporation into a step edge or kink, then attachment to a kink, attachment to a straight step edge, and finally adsorption on a terrace. Elements that form stronger bonds with the host are therefore stabilised more strongly when their coordination increases, giving rise to the characteristic U-shaped trends in reaction energies, with central TMs exhibiting the largest stabilisation upon incorporation. For early TMs, the energy gained by forming additional bonds is (partly) offset by steric strain arising from their larger atomic size compared with the more compact coinage metal lattice. Late TMs, although similar in size to the host, form weaker bonds with host atoms,^17^ so that increasing the number of neighbours yields only limited stabilisation. Moreover, incorporation necessarily moves a host atom from a highly coordinated lattice position to a less coordinated surface site, and the resulting loss of coordination reduces the overall exothermicity of the reaction. As long as the hetero adatom forms stronger bonds with the host than the host does with itself, incorporation remains exothermic. However, when the hetero adatom forms weaker bonds, for example Ag incorporated into Cu, the reaction can become endothermic.

In contrast to the pronounced U-shaped reaction energies, the reaction barriers associated with the different incorporation pathways exhibit more diverse trends across the 4d series. Overall, three patterns emerge: (1) an attenuated U-shape, (2) a gradual increase from early to late TMs, and (3) no discernible dependence on the dopant element. U-shaped trends tend to arise when the moving host atom remains in close contact with the replacing adatom in the transition state (TS), as in the *terrace pop-in*, *terrace slide-in*, *step drop-in*, *step-adjacent drop-in*, and *kink lift-in* mechanisms illustrated in Fig. 2. Late TMs, being more inert, are less able to stabilise the detaching host atom in the TS, which leads to higher barriers, whereas central TMs stabilise such TSs more effectively through stronger bonding. For very early TMs, the larger atomic size can destabilise the TS when the dopant is in close proximity to the moving host atom and the confined structure of the TS cannot accommodate it efficiently. Barriers that increase gradually from early to late TMs are observed for the *step lift-in* mechanism. In this case, the detaching host atom is better stabilised by larger early TMs, while the progressive weakening of bonding toward late TMs leads to increasing barriers. Finally, no clear periodic trends are observed when the TS involves substantial detachment of the host atom before new bonds to the adatom can form, as in the *step pop-in* and *near-kink pop-in* mechanisms, or when incorporation from above into step edges or kinks proceeds *via* nearly barrierless *drop-in* pathways. For these mechanisms, barriers show only a slight increase for the latest TMs, reflecting their weaker bonding to the host surface.

These periodic trends are consistent across both host metals. However, barriers on Cu surfaces are slightly higher, reflecting the stronger Cu–Cu bonds that need to be broken during the detachment of a host atom accompanying adatom incorporation. Reactions are also slightly less exothermic for Cu, which can be attributed to the energy penalty of breaking Cu–Cu bonds and to the shorter interatomic distances compared with Ag. Because the incorporated dopants are 4d TMs, which are larger than the 3d Cu atoms but more similar in size to Ag, the size mismatch in Cu introduces additional strain, thereby reducing the energetic stabilisation gained upon incorporation. Consequently, if a stronger thermodynamic driving force for dopant incorporation into Cu surfaces is desired, smaller 3d TM dopants are expected to be more favourable candidates due to their better size match with the Cu lattice, although spin effects may also influence the energetics. Conversely, when dopant segregation into the bulk is a concern, combining a Cu host, with its stronger host–host bonding and smaller lattice spacing, with larger 4d TM dopants may be advantageous.

Taken together, the periodic trends in reaction energies and barriers provide a detailed understanding of how adatom properties influence individual incorporation mechanisms, but they do not, on their own, determine how dopants actually enter the surface during SAA formation. To resolve which pathways operate in practice, it is also necessary to consider how frequently adatoms reach the relevant reactant states in addition to the intrinsic reaction barriers.

### Step edges and kinks dominate dopant incorporation

Assuming an adatom is deposited on a terrace site, which is the most common surface type, it will first diffuse rapidly, since terrace diffusion is much more facile than any direct incorporation process into the terrace. As a result, adatoms typically explore large areas of the surface and encounter defect sites before terrace incorporation can occur. Although thermal fluctuations might in principle generate short-lived local reconstructions forming transient defects, their formation would be associated with a substantial energetic penalty^74,75^ and is therefore expected to be rare compared to incorporation at pre-existing defects. At elevated temperatures, however, the *terrace slide-in* pathway may become accessible for early TMs on pristine terraces, as the barriers can decrease to about 36 kJ mol^−1^ for Nb on Ag surfaces. Barriers of this magnitude are not insurmountable at room temperature and above. However, because terrace diffusion barriers are extremely low, the *terrace slide-in* mechanism competes with more facile processes discussed below. Even a barrier of 36 kJ mol^−1^ is therefore relatively high in comparison, as barrier differences of this size correspond to rate ratios of several orders of magnitude. Nevertheless, in absolute terms, these barriers remain moderate and, unlike in catalytic cycles, incorporation needs to occur only once to generate an embedded dopant that can have a long-term impact on the surface structure and reactivity. Thus, even if less probable than other pathways, both transient defect formation^74,75^ on terraces with subsequent adatom incorporation and concerted mechanisms^54,76^ deserve more attention in mechanistic studies than they have received so far.

**Fig. 4:**
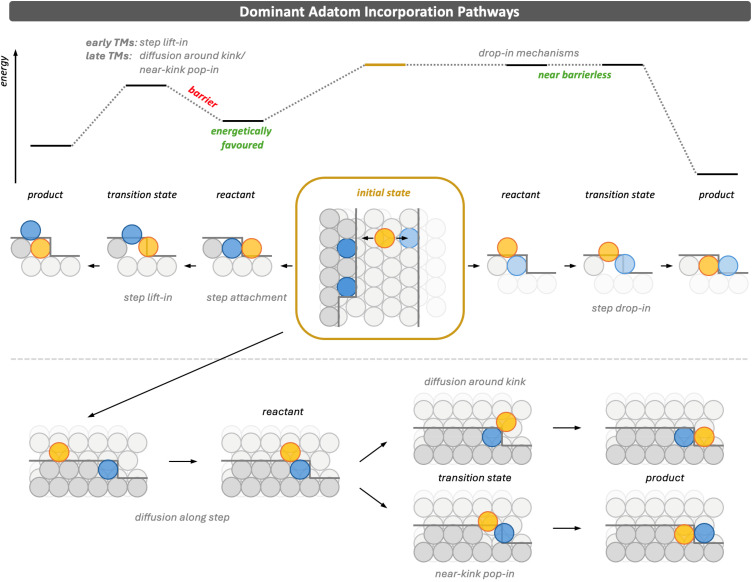
Schematic illustration of the dominant incorporation pathways at step edges and kinks. Adatoms deposited on terraces can diffuse to defects and approach from either the upper or lower terrace. Incorporation from above (top right) proceeds *via* a *drop-in* mechanism. Incorporation from below (top left) follows attachment to the ascending step and subsequent *step lift-in*, or diffusion along the step followed either by *diffusion around kink* or incorporation *via* the *near-kink pop-in* mechanism (bottom). Schematic energy profiles are shown above the structural representations. Adatoms and dopants are shown in orange, moving host atoms in blue, and host atoms in shades of grey. Simulation cell sizes are provided in Section S1 of the SI.

All step edges have an ascending and a descending side. Hence, diffusing adatoms have equal probability to approach a step from the lower or the upper terrace.^48^ Each of these approaches can initiate one of the dominant pathways illustrated in Fig. 4. Thermodynamically, attachment to a step from below (left) is strongly favoured, and attachment to a kink (bottom) is even more stabilising.

The barriers for *diffusion along steps* are below 50 kJ mol^−1^, with the highest value found for the late TM Rh on Ag. For early TMs, these barriers can be as low as 12 kJ mol^−1^, for example for Y on Cu. This diffusion competes with incorporation into the step *via* the *step lift-in* mechanism, whose barriers similarly increase from early to late TMs, ranging from 32 kJ mol^−1^ for Zr on Cu up to about 100 kJ mol^−1^ for Pd on Cu. Although incorporation into the step is therefore possible, particularly for early and central TMs, the barriers are considerable. Crucially, however, for all but the latest TMs, the barrier for incorporation from below is still smaller than the energy required to detach from a step edge and even more so from a kink. This means that once an adatom has attached, it can either remain at the step, diffuse along it until it encounters a kink, or incorporate into the step.

When a dopant diffuses along a step and approaches a kink, two competing processes become possible. Intuitively, the dopant may diffuse around the kink, thereby propagating the step and forming the new kink atom. Alternatively, it can follow the *near-kink pop-in* mechanism, in which the dopant increases its coordination with host atoms by displacing the kink atom, which shifts by one lattice position and thus also grows the step. The relative importance of these pathways depends on the dopant element. While the barrier for *step lift-in* increases from early to late TMs, as shown in Fig. 3, the barrier for *near-kink pop-in* shows little variation across the series, and diffusion around the kink exhibits an inverse U-shaped trend. As a result, very early TMs preferentially incorporate *via step lift-in*, whereas later TMs are more likely to diffuse along the step and either continue around the kink, thereby attaching to it, or incorporate *via* the *near-kink pop-in* mechanism.

When an adatom approaches a step edge from the upper terrace (right), incorporation is generally much easier than incorporation from below. Consistent with previous studies,^30,48,58,62,64^ the *step drop-in*, *near-kink drop-in*, and *kink drop-in* mechanisms, in which the adatom replaces a host atom directly in the step edge or at a kink, are (nearly) barrierless for most dopant elements, except for the very latest TMs. Once the adatom is sufficiently close to the defect, it can form bonds with the undercoordinated host atoms at the step or kink, while fewer bonds of the displaced host atom must be broken. This stabilises the transition state and enables essentially immediate incorporation.

Because adatoms perform a random walk across terraces, approach from above is just as likely as approach from below, and incorporation from above can therefore occur as an irreversible step, analogous to the often irreversible attachment of adatoms to defects from the lower terrace. Although the reactant state for incorporation from above is thermodynamically much less stable, the decisive factor is how the adatom arrives at the defect rather than the relative energies of the initial states.

An important exception to this general picture is Ag. For Ag adatoms on Cu, the energy required for detachment from a step edge is only 44 kJ mol^−1^, which is lower than the incorporation barrier of 83 kJ mol^−1^. This implies that attachment of Ag to a step edge can be reversible, in contrast to the behaviour of most other 4d TMs. In addition, the Ehrlich–Schwoebel barrier for diffusion over a step is relatively small, 42 kJ mol^−1^, and identical to the lowest barriers for incorporation from above *via* the *near-kink drop-in*. Together, these properties indicate that Ag adatoms remain unusually mobile: they can detach from step edges, hop between terraces, and do not necessarily become trapped or promptly incorporated. This enhanced mobility is consistent with experimental observations for Ag adatoms on Cu.^48^

Our results indicate that adatom incorporation occurs predominantly at step edges and kinks. This raises the question of how dopants can be experimentally observed in terrace-like regions that appear away from defects.^43,47,48,77^ Two explanations are conceivable. Embedded dopants could either diffuse from defect sites into terraces after incorporation, or they could remain near their incorporation site while steps propagate, generating new terrace areas around the immobile dopants.

To assess these possibilities, we examine whether embedded dopants can migrate within the surface. If dopant diffusion were facile, both mechanisms could contribute; if not, step propagation would be the more likely explanation. In contrast to the rapid diffusion of adatoms on terraces, the diffusion of dopants embedded in defect-free terraces is highly unfavourable. Our attempts to locate transition states for direct concerted motion of dopant and host atoms were either unsuccessful or resulted in barriers that were too high to be overcome. We therefore considered potentially more favourable diffusion pathways involving (temporary) vacancies.

The *adatom–vacancy pair formation* mechanism reported in Section S2 of the SI starts with a dopant embedded in a terrace that pushes a neighbouring host atom onto the surface, thereby moving the dopant by one lattice position. The barriers for this process are high, ranging from 127 kJ mol^−1^ for a Cu dopant in an Ag surface to 220 kJ mol^−1^ for Ru in a Cu surface. Although transition states can be located for this mechanism, the barriers are too high to provide a viable pathway for dopant diffusion in terrace-like regions.

In addition to describing dopant diffusion, this mechanism also corresponds to vacancy formation next to a dopant. The reaction is highly endothermic, with reaction energies consistently exceeding 100 kJ mol^−1^ across the dopant series, as shown in Section S2 of the SI. Although vacancy formation is therefore unlikely, the presence of such a vacancy would provide a reactant state for vacancy-mediated dopant diffusion, a potentially more favourable diffusion pathway. We therefore consider this scenario as a lower-bound estimate for the minimum possible diffusion barriers.

Specifically, two such mechanisms are investigated and illustrated in Fig. 1. Assuming that a dopant becomes incorporated at a defect, the first diffusion step into terrace-like regions involves migration of a dopant from a step edge into a neighbouring vacancy *via* the *vacancy-mediated dopant diffusion from step* mechanism. This process exhibits moderate barriers, increasing from early to late TMs and reaching 33 kJ mol^−1^ for Pd in Ag, indicating that dopants located directly in a step edge could diffuse into an existing vacancy. However, to explain dopants in terraces distant from defects, further diffusion within terrace regions would be required. Even in the presence of a vacancy, the barriers for the *vacancy-mediated dopant diffusion in terraces* mechanism remain substantial, reaching up to 113 kJ mol^−1^ for the central TM Ru in Ag, with somewhat lower values for very early and late TMs, for example 44 and 52 kJ mol^−1^ for Zr and Ag in Cu, respectively.

Taken together, the large energetic penalty associated with vacancy formation next to a dopant and the considerable barriers for dopant diffusion *via* the mechanisms investigated here, even in the presence of adjacent vacancies, indicate that once incorporated, most dopants are effectively immobile. Post-incorporation diffusion is therefore unlikely to account for dopants observed in terrace-like regions. Instead, their presence away from defects is most plausibly explained by step propagation following incorporation.

Independent of the dominant pathway by which an adatom becomes incorporated, the dopant is initially placed near the defect. Continued incorporation therefore leads to a locally increased dopant concentration in this region. As the step grows, this dopant accumulation manifests as a dopant-rich brim, in agreement with experimental observations.^41,43,47,48,78,79^ Thus, depending on the dopant elements, the diffusion and incorporation of subsequently deposited adatoms may be affected by the brim.

### Embedded dopants can anchor diffusing adatoms when interactions are attractive or, when repulsive, form exclusion zones that suppress incorporation pathways

**Fig. 5:**
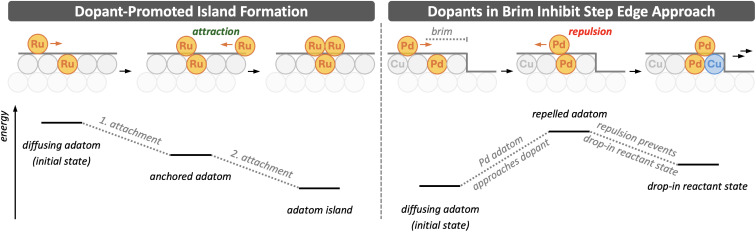
Schematic illustration of the effects of embedded dopants on adatom diffusion and incorporation. Left: Dopant-promoted island formation, where attractive adatom–dopant interactions immobilise adatoms and facilitate subsequent adatom attachment and island growth. Right: Brim-induced suppression of incorporation, where repulsive interactions prevent adatoms from approaching step edges from above and inhibit *drop-in* pathways. Schematic energy profiles are shown beneath the structures. Adatoms and dopants are shown in orange, moving host atoms in blue, and host atoms in grey (upper layer) and light grey (lower layer). The direction of adatom diffusion is indicated by orange arrows. Simulation cell sizes are provided in Section S1 of the SI.

The mechanistic picture developed so far does not account for how dopants that are already embedded in the surface influence the diffusion and incorporation of subsequently deposited adatoms. Depending on the dopant element, embedded dopants can either attract or repel additional adatoms, and, as we will show, these interactions can strongly affect whether adatoms incorporate, remain mobile, or form adatom islands. Fig. 5 illustrates these effects for both attractive and repulsive adatom–dopant interactions.

Very late TMs, such as Pd, do not form stabilising bonds with each other.^17^ This behaviour has important consequences and explains surprising experimental observations for Pd in Cu.^42,47,79^ Experiments show that the concentration of Pd dopants is locally increased in the upper terrace adjacent to a step edge, forming a Pd-rich brim. The width of this brim, and thus the total amount of incorporated Pd, correlates with the area of the lower terrace connected to the step edge, but not with the area of the upper terrace. This observation indicates that incorporation proceeds predominantly *via* pathways in which adatoms approach the step from below, while incorporation from above is effectively suppressed.

This apparent contradiction to our mechanistic picture, which suggests that incorporation should occur from both the upper and lower terrace, can be rationalised by the repulsive interaction between Pd adatoms and embedded Pd dopants. At low dopant concentrations, adatom diffusion across terraces is only weakly perturbed, as adatoms can diffuse around isolated dopants. However, once a brim with a high local Pd concentration has formed, crossing this region becomes energetically unfavourable. The diffusion barrier for crossing near a Pd dopant is considerable, 33 kJ mol^−1^, compared to only 3 kJ mol^−1^ on pure Cu. As a result, adatoms no longer perform a random walk but avoid Pd-rich regions, effectively creating an exclusion zone behind such areas. Because this repulsion prevents adatoms from entering and crossing the dopant-rich upper-terrace brim, the reactant state required for low-barrier incorporation from above cannot form. Consequently, incorporation proceeds *via* attachment from below, consistent with the experimental observations.

In contrast to the repulsive behaviour observed for Pd, attractive adatom–dopant interactions can immobilise diffusing adatoms. This is the case for most of the investigated dopant elements: the attraction is weakest, or even slightly repulsive, for very early and late TMs and strongest for central TMs. This is shown in Section S3 in the SI. Ru serves as a representative example of such attractive interactions. While diffusion of a Ru adatom across a clean terrace is very facile, with barriers below 5 kJ mol^−1^, its attachment to an embedded Ru dopant is strongly exothermic, releasing 135 kJ mol^−1^ on Cu and up to 236 kJ mol^−1^ on Ag. This strong stabilisation immobilises the adatom. Such trapped adatoms may promote the formation of adatom islands on terraces, as further indicated by the even more exothermic attachment of a second Ru adatom to the first Ru adatom immobilised at the dopant site, with an attachment energy of −152 kJ mol^−1^. These reactions, illustrated in Fig. 5, highlight how dopants can stabilise undercoordinated surface species, in line with recent findings.^67^

Because these adatoms no longer diffuse, they cannot reach step edges or kink sites to incorporate *via* the dominant pathways. We therefore also investigate incorporation on a terrace adjacent to the dopant *via* the concerted *terrace slide-in* mechanism, which exhibits the lowest barriers on pristine host terraces. The barrier for incorporating a Ru adatom next to an embedded Ru dopant is 91 kJ mol^−1^, substantially higher than on the pristine host surface, 59 kJ mol^−1^. This behaviour can be rationalised by the bonding changes occurring in the transition state. The Ru–Ru bond formed upon adsorption of the Ru adatom at the embedded Ru dopant remains intact, so no additional stabilisation is gained, while a dopant–host bond must be broken to accommodate the incoming adatom as it becomes incorporated into the surface adjacent to the dopant. Since Ru forms stronger bonds with Cu than Cu does with itself, breaking a Ru–Cu bond incurs a larger energetic penalty than breaking a Cu–Cu bond on a pure host surface, leading to the increased incorporation barrier. As a consequence, although Ru dopants strongly attract and immobilise Ru adatoms on the surface, the formation of embedded Ru dimers or larger embedded dopant clusters may be self-inhibiting. Reaction energies for adatom attachment to embedded dopants for all investigated dopants are reported in Section S3 in the SI.

## Conclusion

This work compiles existing mechanistic understanding of individual adatom incorporation pathways on metal surfaces and builds upon it to provide a broad and coherent picture of how embedded active sites form in single-atom alloys. By treating all relevant processes within a single, consistent DFT framework, we identify and rationalise periodic trends across the 4d transition metal series, obtain directly comparable reaction energetics that can serve as input for kinetic Monte Carlo (kMC) simulations of step growth and surface restructuring, and establish which incorporation pathways dominate. In line with conventional understanding, direct incorporation into defect-free terrace sites is generally unfavourable. However, a concerted three-atom mechanism may provide a viable incorporation route at elevated temperatures or on sufficiently extended terraces.

Surface defects, in contrast, enable efficient adatom incorporation. Both step edges and kink sites facilitate (almost) barrierless incorporation for most TMs when approached from above, with step edges alone already providing low-barrier pathways. Attachment to step edges, and particularly to kink sites, from the lower terrace is thermodynamically strongly favoured and effectively irreversible for most TMs. Once attached, adatoms may initiate step growth or incorporate into the step, as incorporation barriers are usually smaller than those for detachment. Ag represents an exception. On Cu surfaces, detachment of Ag adatoms from step edges is easier than incorporation, and the combination of comparatively high incorporation barriers and low barriers for diffusion over steps makes Ag adatoms unusually mobile, allowing them to move between terraces.

Periodic trends reveal that early and central TMs incorporate most readily, whereas very late TMs exhibit higher barriers for the relevant pathways. Overall, Cu hosts display slightly higher incorporation barriers and less exothermic reaction energies than Ag, reflecting stronger host–host bonding and a larger size mismatch with 4d dopants.

Finally, adatom–dopant interactions can significantly modulate diffusion and incorporation. Repulsive interactions, as in Pd-doped Cu, suppress incorporation from above by creating dopant-rich brims that act as exclusion zones, preventing adatoms from reaching the step edge and forming the necessary reactant states. Attractive interactions, exemplified by Ru, immobilise diffusing adatoms at embedded dopants and can promote the formation of anchored adatom islands, stabilising undercoordinated atoms that may be crucial for catalytic activity.

Together, these findings bridge the gap between elementary incorporation mechanisms and experimentally observed synthesis outcomes, providing mechanistic insight into SAA formation and guidance on which surface environments most effectively promote incorporation for different dopant elements, as well as how specific dopant–adatom interactions influence the formation and nature of active sites.

## Conflicts of interest

There are no conflicts to declare.

## Supplementary Material

NR-018-D5NR05517B-s001

NR-018-D5NR05517B-s002

## Data Availability

The data supporting this article, coordinate files, and example inputs are provided as part of the supplementary information (SI). Supplementary information is available. See DOI: https://doi.org/10.1039/d5nr05517b.
