# Supplementary material for: Mechanisms for the formation of active sites in single-atom alloys
Source: Nanoscale. 2026 Apr 13;18(18):9709–22. doi: 10.1039/d5nr05517b (PMC13071885; doi:10.1039/d5nr05517b)
Supplement: NR-018-D5NR05517B-s002 [file NR-018-D5NR05517B-s002.pdf]

Supplementary Information (SI) for Nanoscale.
This journal is © The Royal Society of Chemistry 2026

Supplementary Information: Mechanisms for the Formation of Active Sites in Single-Atom Alloys

Ioannis Karageorgiou, Angelos Michaelides,* and Fabian Berger*

Yusuf Hamied Department of Chemistry, University of Cambridge, CB2 1EW Cambridge, UK

E-mail: am452@cam.ac.uk; fb593@cam.ac.uk

Contents

S1 Computational Details **S-3**

S2 Periodic Trends in Reaction Energies and Barriers **S-5**

S3 Reaction Energies and Barriers for Dopant–Adatom Interactions **S-14**

References **S-17**

S1 Computational Details

Periodic density functional theory (DFT) calculations with the optB86b-vdW functional[S1] are performed as implemented in VASP 6.4.1, using plane wave basis sets for valence electrons and the projector augmented wave (PAW) method for core electrons with standard potentials.[S2–S5] Further, first order Methfessel-Paxton smearing (SIGMA = 0.1), dipole correction, and the following POTCARs are used: Cu: Cu, Ag: Ag, Y: Y_sv, Zr: Zr_sv, Nb: Nb_sv, Mo: Mo_sv, Tc: Tc_pv, Ru: Ru_pv, Rh: Rh_pv, Pd: Pd.

The metal surfaces are modelled using five atomic layers, with the bottom two layers fixed, and a vacuum region of 15 Å. Simulation cell sizes are chosen such that dopants or adatoms are separated by at least two host atoms. For the (111) facet, calculations are carried out in $(3 \times 3 \times 5)$ and $(4 \times 4 \times 5)$ supercells. For the (211) facet, cells containing three atom rows along each lateral direction are used. For the (322) facet, we employ cells with three and five atom rows along the lateral directions. Kink-site models are generated by attaching additional host atoms to the (322) step edge, using simulation cells with four and five atom rows along the lateral directions. All structure files are provided as part of the Supplementary Information.

Unit cell vectors are determined using the equation of state method[S6] as implemented in the Atomic Simulation Environment (ASE), an energy cutoff of 800 eV is used to minimise the influence of volume change on the basis set, and the Brillouin zone is sampled using a Monkhorst-Pack mesh with $31 \times 31 \times 31$ $\mathbf{k}$ points.

For structure optimisations, the plane-wave energy cutoff is set to 400 eV, and the Brillouin zone is sampled using a $5 \times 5 \times 1$ Monkhorst–Pack $\mathbf{k}$-point mesh.[S7] Structures are relaxed using the conjugate-gradient algorithm until all forces are below 0.01 eV/Å.

Transition state structures are first located using the Nudged Elastic Band (NEB) method as implemented in the VTST tools for VASP,[S8,S9] employing 9 images with the initial and final images fixed. NEB optimisations are stopped when all atomic forces are below 0.1 eV/Å. The images with the highest are then interpolated to construct initial guesses for the dimer

method, which is subsequently used to refine the transition state structure.[S10] Dimer calculations are converged when all forces are below 0.01 eV/Å.

S2 Periodic Trends in Reaction Energies and Barriers

Table S1: **Reaction energies** for **diffusion mechanisms** on **Cu surfaces**. Terrace diffusion is defined as diffusion from fcc to hcp adsorption sites. Energies are reported in kJ mol^{-1} for Cu ($3d$) and the series of $4d$ transition metal adatoms. *Dopant diffusion in terrace* and *dopant diffusion from step* correspond to vacancy-mediated mechanisms. Reaction energies correspond to the energy difference between the initial and final states, as defined in the main text.

mechanism	Cu	Y	Zr	Nb	Mo	Tc	Ru	Rh	Pd	Ag
terrace diffusion	1	0	−2	−2	−1	2	3	4	2	0
dopant diffusion in terrace	0	0	0	0	0	0	0	0	0	0
diffusion over step	−60	-	−118	−123	-	−112	−106	−95	−78	−50
step attachment	−55	-	−99	−111	−115	−115	−112	−104	−81	−44
diffusion along step	0	0	0	0	0	0	0	0	0	0
dopant diffusion from step	−47	−54	−85		−104	−104	−99	−84	−59	−34
kink attachment	−79	-	-	−170	−172	−171	−161	−146	−112	−63
diffusion around kink	−24	−39	−50	−58	−62	−59	−53	−44	−31	−18

Table S2: **Reaction barriers** for **diffusion mechanisms** on **Cu surfaces**. Terrace diffusion is defined as diffusion from fcc to hcp adsorption sites. Barriers for attachment to step edges and kink sites are not reported, as they are lower than the terrace diffusion barriers. Energies are reported in kJ mol^{-1} for Cu ($3d$) and the series of $4d$ transition metal adatoms. *Dopant diffusion in terrace* and *dopant diffusion from step* correspond to vacancy-mediated mechanisms. Reaction barriers are defined as the energy difference between the initial and the transition state, as defined in the main text.

mechanism	Cu	Y	Zr	Nb	Mo	Tc	Ru	Rh	Pd	Ag
terrace diffusion	5	0	2	4	4	5	5	4	3	4
dopant diffusion in terrace	66	16	44	70	83	97	103	102	82	52
diffusion over step	49	-	104	126	-	134	123	108	77	42
step attachment	-	-	-	-	-	-	-	-	-	-
diffusion along step	30	12	21	26	32	36	40	42	35	19
dopant diffusion from step	24	4	1	-	4	11	18	26	30	24
kink attachment	-	-	-	-	-	-	-	-	-	-
diffusion around kink	31	31	43	50	53	54	54	51	40	22

Table S3: **Reaction energies** for **diffusion mechanisms** on **Ag surfaces**. Terrace diffusion is defined as diffusion from fcc to hcp adsorption sites. Energies are reported in kJ mol^{-1} for Cu ($3d$) and the series of $4d$ transition metal adatoms. *Dopant diffusion in terrace* and *dopant diffusion from step* correspond to vacancy-mediated mechanisms. Reaction energies correspond to the energy difference between the initial and final states, as defined in the main text.

mechanism	Cu	Y	Zr	Nb	Mo	Tc	Ru	Rh	Pd	Ag
terrace diffusion	1	0	−1	−1	1	2	3	4	3	0
dopant diffusion in terrace	0	0	0	0	0	0	0	0	0	0
diffusion over step	−58	-	−109	−113	−111	−108	−103	−94	−84	−52
step attachment	−54	−77	−100	−107	−107	−107	−110	−102	−81	−48
diffusion along step	0	0	0	0	0	0	0	0	0	0
dopant diffusion from step	−39	−62	−88	−96	−96	−95	−90	−78	−57	−35
kink attachment	−78	-	−147	−158	−160	−157	−151	−138	−110	−69
diffusion around kink	−26	−41	−50	−56	−58	−55	−50	−43	−33	−22

Table S4: **Reaction barriers** for **diffusion mechanisms** on **Ag surfaces**. Terrace diffusion is defined as diffusion from fcc to hcp adsorption sites. Barriers for attachment to step edges and kink sites are not reported, as they are lower than the terrace diffusion barriers. Energies are reported in kJ mol^{-1} for Cu ($3d$) and the series of $4d$ transition metal adatoms. *Dopant diffusion in terrace* and *dopant diffusion from step* correspond to vacancy-mediated mechanisms. Reaction barriers are defined as the energy difference between the initial and the transition state, as defined in the main text.

mechanism	Cu	Y	Zr	Nb	Mo	Tc	Ru	Rh	Pd	Ag
terrace diffusion	7	1	2	3	4	5	5	4	5	5
dopant diffusion in terrace	57	44	77	7	106	110	113	108	86	57
diffusion over step	42	-	98	115	120	118	110	96	65	38
step attachment	-	-	-	-	-	-	-	-	-	-
diffusion along step	33	22	33	38	41	44	47	49	41	24
dopant diffusion from step	21	6	8	-	16	24	29	33	33	26
kink attachment	-	-	-	-	-	-	-	-	-	-
diffusion around kink	33	33	42	43	47	52	-	52	43	25

Table S5: **Reaction energies** for **incorporation mechanisms** on **Cu surfaces**. Energies are reported in kJ mol^{-1} for Cu ($3d$) and the series of $4d$ transition metal adatoms. Reaction energies correspond to the energy difference between the initial and final states, as defined in the main text. For the *step pop-in* mechanism, a high-lying intermediate may exist; when this occurs, the reaction energies are reported separately for the step from the initial state to the intermediate (1) and from the intermediate to the final state (2). The *adatom–vacancy pair formation* mechanism starts with a dopant in a terrace site replacing a neighbouring host atom, which is expelled onto the surface, thereby creating a vacancy next to the dopant.

mechanism	Cu	Y	Zr	Nb	Mo	Tc	Ru	Rh	Pd	Ag
terrace pop-in	1	−24	−109	−161	−184	−190	−169	−125	−47	31
terrace slide-in	1	−30	−119	−145	−163	−162	−150	−116	−51	34
step drop-in	−57	-	−148	−177	−188	−193	−185	−149	−100	−30
step-adjacent drop-in	−55	-	−136	−189	−214	−220	−207	−172	−104	−18
step lift-in	58	36	17	0	−8	−12	−4	17	50	66
step pop-in	0	5	−33	−55	−68	−75	−69	−52	−19	17
step pop-in (1)	75	77	74	-	-	73	74	73	76	74
step pop-in (2)	−75	−72	−107	-	-	−148	−142	−126	−95	−57
near-kink drop-in	−57	-	−139	−180	−197	−198	−185	−150	−99	−30
kink drop-in	−79	-	-	−208	−216	−218	−204	−176	−122	−52
near-kink pop-in	−24	−32	−52	−66	−75	−75	−66	−52	−33	−12
kink lift-in	80	84	46	24	15	15	24	44	71	90
adatom–vacancy pair formation	146	119	131	143	152	158	165	166	158	136

Table S6: **Reaction barriers** for **incorporation mechanisms** on **Cu surfaces**. Energies are reported in kJ mol^{-1} for Cu ($3d$) and the series of $4d$ transition metal adatoms. Reaction barriers are defined as the energy difference between the initial and the transition state, as defined in the main text. For the *step pop-in* mechanism, a high-lying intermediate may exist; when this occurs, the reaction energies are reported separately for the step from the initial state to the intermediate (1) and from the intermediate to the final state (2). The *adatom–vacancy pair formation* mechanism starts with a dopant in a terrace site replacing a neighbouring host atom, which is expelled onto the surface, thereby creating a vacancy next to the dopant.

mechanism	Cu	Y	Zr	Nb	Mo	Tc	Ru	Rh	Pd	Ag
terrace pop-in	136	121	99	96	96	95	98	108	131	149
terrace slide-in	124	79	51	41	42	48	59	80	103	130
step drop-in	38	-	4	0	0	0	0	9	21	46
step-adjacent drop-in	86	-	46	32	27	28	33	44	66	95
step lift-in	97	43	32	38	47	53	64	83	101	-
step pop-in	81	86	79	76	75	74	77	82	86	83
step pop-in (1)	81	86	79	-	-	74	74	73	77	82
step pop-in (2)	6	1	0	-	-	0	4	9	10	8
near-kink drop-in	30	-	-	11	10	10	8	10	14	42
kink drop-in	38	-	-	0	0	8	2	7	22	45
near-kink pop-in	62	49	47	46	46	50	57	62	63	60
kink lift-in	109	137	112	101	95	95	101	113	125	134
adatom–vacancy pair formation	175	142	152	174	192	206	220	220	202	175

Table S7: **Reaction energies** for **incorporation mechanisms** on **Ag surfaces**. Energies are reported in kJ mol^{-1} for Cu ($3d$) and the series of $4d$ transition metal adatoms. Reaction energies correspond to the energy difference between the initial and final states, as defined in the main text. For the *step pop-in* mechanism, a high-lying intermediate may exist; when this occurs, the reaction energies are reported separately for the step from the initial state to the intermediate (1) and from the intermediate to the final state (2). The *adatom–vacancy pair formation* mechanism starts with a dopant in a terrace site replacing a neighbouring host atom, which is expelled onto the surface, thereby creating a vacancy next to the dopant.

mechanism	Cu	Y	Zr	Nb	Mo	Tc	Ru	Rh	Pd	Ag
terrace pop-in	−18	−91	−169	−201	−206	−202	−183	−147	−81	0
terrace slide-in	−3	−76	−146	−176	−179	−173	−158	−126	−72	0
step drop-in	−55	−114	−171	−193	−195	−190	−176	−151	−111	−50
step-adjacent drop-in	−54	−114	−189	−218	−223	−219	−210	−177	−119	−48
step lift-in	39	11	−13	−28	−32	−31	−21	−2	26	49
step pop-in	0	−31	−58	−75	−78	−78	−71	−57	−29	0
step pop-in (1)	59	56	55	-	-	59	63	62	63	58
step pop-in (2)	−59	−87	−113	-	-	−137	−134	−119	−92	−58
near-kink drop-in	−59	−111	−164	−194	−194	−200	−181	−152	−107	−46
kink drop-in	−76	-	−189	−209	−212	−207	−194	−170	−126	−69
near-kink pop-in	−27	−45	−62	−76	−80	−76	−68	−56	−40	−22
kink lift-in	57	36	0	−15	−17	−14	−3	15	43	69
adatom–vacancy pair formation	106	113	118	123	125	127	128	122	121	120

Table S8: **Reaction barriers** for **incorporation mechanisms** on **Ag surfaces**. Energies are reported in kJ mol^{-1} for Cu ($3d$) and the series of $4d$ transition metal adatoms. Reaction barriers are defined as the energy difference between the initial and the transition state, as defined in the main text. For the *step pop-in* mechanism, a high-lying intermediate may exist; when this occurs, the reaction energies are reported separately for the step from the initial state to the intermediate (1) and from the intermediate to the final state (2). The *adatom–vacancy pair formation* mechanism starts with a dopant in a terrace site replacing a neighbouring host atom, which is expelled onto the surface, thereby creating a vacancy next to the dopant.

mechanism	Cu	Y	Zr	Nb	Mo	Tc	Ru	Rh	Pd	Ag
terrace pop-in	100	78	72	77	80	80	83	90	107	112
terrace slide-in	103	53	38	36	37	42	51	65	86	108
step drop-in	28	16	1	1	0	1	3	10	16	35
step-adjacent drop-in	68	48	28	24	24	25	25	35	54	76
step lift-in	76	31	36	42	48	53	64	76	87	86
step pop-in	66	66	64	65	65	65	65	69	73	67
step pop-in (1)	66	66	64	-	-	65	65	64	67	67
step pop-in (2)	5	1	0	-	-	0	1	6	10	9
near-kink drop-in	0	0	0	0	0	0	0	1	10	31
kink drop-in	28	-	0	0	0	0	2	7	18	35
near-kink pop-in	46	36	42	38	40	46	50	52	53	51
kink lift-in	72	89	73	65	63	64	70	78	89	96
adatom–vacancy pair formation	127	131	158	177	185	192	196	187	168	147

S3 Reaction Energies and Barriers for Dopant–Adatom Interactions

The reaction energy for the diffusion of a Pd adatom from an fcc to an hcp site when crossing an embedded Pd dopant on a Cu surface is 2 kJ mol^{-1}, with a corresponding barrier of 33 kJ mol^{-1}. On a pristine Cu surface, the reaction energy is identical, 2 kJ mol^{-1}, but the barrier is much lower at 3 kJ mol^{-1}. This increase in the diffusion barrier indicates that Pd adatoms avoid diffusing across embedded Pd dopants, corroborating the repulsive interaction between Pd atoms.

In contrast, elements that exhibit attractive interactions, such as Ru, the energy released when a Ru adatom attaches from a pure host terrace site to an embedded Ru dopant is -135 kJ mol^{-1} on Cu and between -232 and -236 kJ mol^{-1} on Ag, indicating strong immobilisation of the Ru adatom at the dopant site. However, the reaction energy for incorporating a Ru adatom next to an embedded Ru dopant via the *terrace slide-in* mechanism on Cu is -69 kJ mol^{-1}, which is substantially less exothermic than the incorporation of the first Ru adatom into a pristine terrace site, -150 kJ mol^{-1}, following the same mechanism. The barrier for this incorporation pathway adjacent to an embedded Ru dopant on Cu is 91 kJ mol^{-1}, which is 32 kJ mol^{-1} higher than the barrier for incorporation of the first Ru atom on a pristine Cu terrace, 59 kJ mol^{-1}, following the same *terrace slide-in* mechanism. This indicates that although incorporation of a second Ru atom next to the dopant is thermodynamically favourable, it is associated with a substantial kinetic barrier, even higher than the incorporation barrier for the *terrace slide-in* mechanism on the pristine surface. Consequently, the formation of embedded dopant clusters may be kinetically hindered, whereas the immobilisation of Ru adatoms at embedded Ru dopants, and the subsequent formation of adatom islands, is thermodynamically favoured. The attachment of a second Ru adatom to a Ru adatom already anchored at an embedded Ru dopant, as illustrated in Figure 5 of the main text, is indeed highly exothermic, releasing -152 kJ mol^{-1}.

Although Ru serves only as a representative example, most investigated dopant elements exhibit exothermic attachment of adatoms to embedded dopants. Table S9 reports the reaction energies for the attachment of an adatom from a pristine host terrace site to an embedded dopant of the same element together with the corresponding dopant–adatom distances. Clear periodic trends are observed both in the reaction energies and in the dopant–adatom distances. For central TMs the distances are short and the attachment energies strongly exothermic, indicating strong attractive bonding interactions. In contrast, for early and late TMs the distances can be considerably larger and attachment becomes less favourable. Consequently, the optimised product state does not always correspond to an adatom directly bonded to the dopant. For example, in the case of Pd or Ag in Cu the dopant–adatom distance can exceed 300 pm, indicating that the adatom remains in the vicinity of the dopant but occupies a hollow site formed solely by Cu atoms rather than forming a direct bond with the dopant. The behaviour of Pd and Ag in Cu indicates a repulsive interaction between the two atoms, consistent with the increased diffusion barrier for Pd adatoms crossing embedded Pd dopants in Cu discussed above. Thus, the reported reaction energies should only be interpreted as the energy of direct adatom–dopant bond formation when the dopant–adatom distance is sufficiently short.

Table S9: Attachment of an adatom to an hcp or fcc site adjacent to the embedded dopant, as shown in Figure 5 in the main text. Note that the original classification of each site may not correspond to the location of the adatom after the geometry optimisation process. E_{attach} is the reaction energy for an adatom moving from a host terrace site to a dopant embedded in the surface, in kJ mol^{-1}. d_{dop} is the distance between the embedded dopant and the attached adatom, in pm.

mechanism	Cu	Y	Zr	Nb	Mo	Tc	Ru	Rh	Pd	Ag
E_{attach} on Cu_{hcp}	0	−1	−28	−153	−286	−261	−135	−18	−2	−5
d_{dop} on Cu_{hcp}	239	421	329	222	209	210	221	244	327	317
E_{attach} on Cu_{fcc}	0	0	−29	−155	−283	−258	−135	−17	3	−4
d_{dop} on Cu_{fcc}	238	428	324	222	209	210	221	245	304	315
E_{attach} on Ag_{hcp}	−18	−25	−113	−348	−484	−412	−236	−74	−7	0
d_{dop} on Ag_{hcp}	233	411	245	214	203	205	215	235	257	273
E_{attach} on Ag_{fcc}	−18	−23	−114	−348	−481	−409	−233	−70	−7	0
d_{dop} on Ag_{fcc}	233	413	245	214	203	205	215	235	257	273

References

(S1) Klimeš, J.; Bowler, D. R.; Michaelides, A. Van der Waals density functionals applied to solids. *Phys. Rev. B* **2011**, *83*, 195131.

(S2) Kresse, G.; Hafner, J. Ab initio molecular dynamics for liquid metals. *Phys. Rev. B* **1993**, *47*, 558.

(S3) Kresse, G.; Furthmüller, J. Efficiency of ab-initio total energy calculations for metals and semiconductors using a plane-wave basis set. *Comput. Mater. Sci.* **1996**, *6*, 15.

(S4) Kresse, G.; Furthmüller, J. Efficient iterative schemes for ab initio total-energy calculations using a plane-wave basis set. *Phys. Rev. B* **1996**, *54*, 11169.

(S5) Kresse, G.; Joubert, D. From ultrasoft pseudopotentials to the projector augmented-wave method. *Phys. Rev. B* **1999**, *59*, 1758.

(S6) Alchagirov, A. B.; Perdew, J. P.; Boettger, J. C.; Albers, R.; Fiolhais, C. Reply to "Comment on 'Energy and pressure versus volume: Equations of state motivated by the stabilized jellium model'". *Phys. Rev. B* **2003**, *67*, 026103.

(S7) Monkhorst, H. J.; Pack, J. D. Special points for Brillouin-zone integrations. *Phys. Rev. B* **1976**, *13*, 5188.

(S8) Henkelman, G.; Uberuaga, B. P.; Jónsson, H. A climbing image nudged elastic band method for finding saddle points and minimum energy paths. *J. Chem. Phys.* **2000**, *113*, 9901.

(S9) Henkelman, G.; Jónsson, H. Improved tangent estimate in the nudged elastic band method for finding minimum energy paths and saddle points. *J. Chem. Phys.* **2000**, *113*, 9978.

(S10) Henkelman, G.; Jónsson, H. A dimer method for finding saddle points on high dimensional potential surfaces using only first derivatives. *J. Chem. Phys.* **1999**, *111*, 7010.
